# Expression from DIF1-motif promoters of *hetR* and *patS* is dependent on HetZ and modulated by PatU3 during heterocyst differentiation

**DOI:** 10.1371/journal.pone.0232383

**Published:** 2020-07-23

**Authors:** Yaru Du, He Zhang, Hong Wang, Shuai Wang, Qiqin Lei, Chao Li, Renqiu Kong, Xudong Xu

**Affiliations:** 1 State Key Laboratory of Freshwater Ecology and Biotechnology, Institute of Hydrobiology, Chinese Academy of Sciences, Wuhan, Hubei, China; 2 University of Chinese Academy of Sciences, Beijing, China; 3 Key Laboratory of Algal Biology, Institute of Hydrobiology, Chinese Academy of Sciences, Wuhan, Hubei, China; Imam Abdulrahman Bin Faisal University, SAUDI ARABIA

## Abstract

HetR and PatS/PatX-derived peptides are the activator and diffusible inhibitor for cell differentiation and patterning in heterocyst-forming cyanobacteria. HetR regulates target genes via HetR-recognition sites. However, some genes (such as *patS*/*patX*) upregulated at the early stage of heterocyst differentiation possess DIF1 (or DIF^+^) motif (TCCGGA) promoters rather than HetR-recognition sites; *hetR* possesses both predicted regulatory elements. How HetR controls heterocyst-specific expression from DIF1 motif promoters remains to be answered. This study presents evidence that the expression from DIF1 motif promoters of *hetR*, *patS* and *patX* is more directly dependent on *hetZ*, a gene regulated by HetR via a HetR-recognition site. The HetR-binding site upstream of *hetR* is not required for the autoregulation of *hetR*. PatU3 (3′ portion of PatU) that interacts with HetZ may modulate the expression of *hetR*, *hetZ* and *patS*. These findings contribute to understanding of the mutual regulation of *hetR*, *hetZ-patU* and *patS*/*patX* in a large group of multicellular cyanobacteria.

## Introduction

Cyanobacteria were the first group of microorganisms that performed oxygenic photosynthesis [1, 2]. In the early earth environment, nitrogen nutrient was a limiting factor for propagation of microbes. Under this selective pressure, *nif* genes spread among bacteria, and some cyanobacteria acquired the N_2_ fixation capability. With the rise of atmospheric oxygen, certain filamentous species developed the capability to form specialized N_2_-fixing cells, called heterocysts, to protect nitrogenase from inactivation by oxygen [3–5]. Nowadays, heterocyst-forming cyanobacteria contribute significantly to nitrogen fixation in the earth’s biosphere [6–8]. In species from different genera of heterocyst-forming cyanobacteria, heterocysts are differentiated at one end, two ends, or intercalary positions of filaments [9]. *Anabaena* sp. PCC 7120 (hereafter *Anabaena* 7120) was derived from a species that produces semi-regularly spaced single heterocysts along non-branched filaments in response to nitrogen stepdown. It is the most often used model strain for molecular studies on heterocyst-related topics [10]. Other species used in such studies include *Anabaena variabilis* [11, 12], *Nostoc punctiforme* [13, 14], *Nostoc ellipsosporum* [15], etc.

Heterocyst differentiation and pattern formation largely depend on the key regulator HetR [16] and RGSGR-containing peptides, which are derived from PatS [17, 18], PatX [19] or HetN [20], representing an example of the most ancient activator-inhibitor (reaction-diffusion) patterning processes [21–23]. In *Anabaena* 7120, PatS is the main source of morphogen for de novo pattern formation [18], while HetN is required for maintenance of the pattern [24]. HetR is the only known target of RGSGR-containing peptides [25], and it binds to consensus recognition sites upstream of *hetP* [26, 27], *hetZ* [28] and several other genes, including its own encoding gene [29–31]. Among these genes, *hetZ* is involved in control of heterocyst differentiation at an early stage [32], and *hetP* is required for commitment to heterocyst differentiation [33]. *hetZ* and *hetP* functionally overlap with each other, and co-expression of these two genes was shown to restore heterocyst formation in *hetR*-minus mutants [34]. In a different substrain of *Anabaena* 7120, expression of *hetZ* alone restored heterocyst formation in a *hetR*-deletion mutant [35]. The variable requirement for *hetP* expression may depend on differences in genetic backgrounds of substrains [36]. *hetP* and *hetZ* are both upregulated in differentiating cells, as a result of the accumulation of HetR [26, 28]. *patS* is also upregulated in differentiating cells [17], but no consensus recognition site for HetR is present in the sequence upstream of *patS*.

Immediately downstream of *hetZ* in many filamentous cyanobacteria is a gene called *patU*; these two genes, together with *hetR*, are listed among the core set of genes for filamentous species [32, 37]. In *Anabaena* 7120, *patU* is split into *patU5* and *patU3* [32]. *hetZ* and *patU3* play opposite roles in heterocyst differentiation: *hetZ* promotes, while *patU3* inhibits [32].

Before the consensus HetR-recognition sequence was identified, DIF^+^ (later called DIF1) motif (TCCGGA) had been bioinformatically identified in sequences upstream of *hetR* and several other genes in *Anabaena* 7120 [38]. The role of DIF1 motif in heterocyst-specific expression was shown with the promoter of *nsiR* (a heterocyst-specific non-coding RNA) [38] and a synthetic minimal promoter [39]. More recently, the DIF1 motif was proposed as a consensus regulatory sequence (centered at -35 region) for *patS* and *patX* in heterocyst-forming cyanobacteria [19]. However, there are two questions to be answered. (1) What is the role of the predicted DIF1 motif promoters in upregulated expression of *hetR*, *patS* and *patX*? This must be examined experimentally. In particular, HetR-recognition site and DIF1 motif are both present upstream of *hetR*. (2) Which of HetR, HetZ and HetP is required for the regulation of DIF1-motif promoters? In *Anabaena* 7120, deletion of *hetZ* blocked the induced expression of *hetR*, *hetP* and *patS*, whereas *hetP* showed no effects on these genes [35]. This result excluded HetP as the factor for inducing the expression of *hetR* and *patS*; however, because *hetR* was not expressed in the *hetZ* mutant, which of HetR and HetZ is required for the upregulated expression of *hetR* and *patS* remained unclear. Earlier, the expression from DIF1 motif promoters had been shown to be dependent on a functional *hetR* [38, 40], but *hetZ* was not expressed in the *hetR* mutant either.

To elucidate the role of HetR and HetZ in control of DIF1 motif promoters during heterocyst differentiation, it is necessary to produce heterocysts without HetR or HetZ. In this study, we tested the expression from P_*hetR*_ and P_*patS*_ in heterocysts without HetR and the role of DIF1 motif in expression of *hetR* and *patS*. We found that HetZ plays a more direct role in control of these promoters than HetR and that the expression of *hetR* and *patS* is mainly dependent on the DIF1-motif promoter sequences. In addition, PatU3 that interacts with HetZ may modulate the expression of *hetR*, *hetZ* and *patS*.

## Materials and methods

### General

*Anabaena* 7120 and derivatives (S1 Table) were cultured in BG11 medium in the light of 30 μE m^-2^ s^-1^ on a rotary shaker. Erythromycin (5 μg ml^-1^), neomycin (20 μg ml^-1^) or spectinomycin (10 μg ml^-1^) was added to the medium as appropriate. For nitrogen stepdown, *Anabaena* 7120 grown in BG11 (OD_730_, 0.7~0.9) was collected by centrifugation, washed 3 times with BG11_0_ (without nitrate) and resuspended in the same medium for 24 or 48 hours as indicated.

### Microscopic observations

Microscopy was performed as previously described [41]. Photomicrographs were captured using an Olympus BX41 microscope (Olympus Corp., Tokyo, Japan) equipped with a JVC 3 CCD colour video camera (TK-C1381) (Victor Company of Japan Ltd, Tokyo, Japan). The GFP fluorescence was observed using a Sapphire GFP filter set (Exciter D395/40, dichroic 425DCLP, and emitter D510/40) (Chroma Technology Corp., Brattleboro, USA); autofluorescence was observed using the red long pass WG fluorescence cube (BP 510–550, BA590) from Olympus.

### Construction of plasmids and *Anabaena* strains

Plasmid construction processes are described in S1 Table in the supplemental materials. DNA fragments cloned by PCR were confirmed by sequencing.

Plasmids were introduced into *Anabaena* 7120 and mutants by conjugation [42]. Homologous double-crossover recombinants were generated based on positive selection with *sacB* [43]. The complete segregation of mutants was confirmed by PCR. *Anabaena* strains and primers are listed in S1 Table.

### Transcription analyses

Total RNA was extracted using Trizol reagent (Invitrogen, Carlsbad, USA), and the residual DNA was removed with DNase RQ1 (Promega, Madison, USA). Reverse transcription was performed with the PrimeScript reverse transcription system (Takara, Dalian, China). RT-qPCR analyses were conducted as we described before [34]. *rnp*B (RNase P subunit B) was used as the internal control. PCR primers (indicated with ‘RT’ in name) are listed in S1 Table. Data are means ± SD produced from 3 technical or biological repeats as indicated.

Promoter activities were visualized using *gfp* (green fluorescence protein) as the reporter gene. Relative copy numbers of zeta- or pDU1-based plasmids (relative to *rnpB*) were evaluated by quantitative PCR as described in the reference [31] using primers gfp-1/gfp-2, pDU1-1/pDU1-2 and rnpB-1/rnpB-2 listed in S1 Table.

### Rapid Amplification of cDNA Ends (RACE)

cDNA was synthesized with the SMART RACE cDNA amplification kit (Clontech, TaKaRa Bio., Otsu, Japan) using random primers. The 5′ end DNA fragments were generated by nested PCR as described by Zhang et al. [32], using universal primer/hetR-race-1 and nested universal primer/hetR-race-2 as the primers for 2 rounds of PCR. The universal primer and nested universal primer were provided with the SMART RACE cDNA amplification kit; hetR-race-1 and hetR-race-2 are listed in S1 Table. Transcription start points were determined based on sequencing of RACE products. Two biological repeats showed similar results.

### Western blot analysis

*Anabaena* 7120 was deprived of fixed nitrogen for 24 h, harvested by centrifugation, washed with 20 mM Tris-HCl (pH 8.0) containing 1 mM PMSF and resuspended in the same buffer. Cells were broken with a French press (SCIENTZ, Ningbo, China) at 240 MPa (cell pressure) and centrifuged at 12,000 × g for 15 min. The supernatant was used as cell extracts for the Western blot analysis.

Proteins were separated by 12% SDS-PAGE and electro-blotted onto NC filters. HetR and HetZ were detected with rabbit antiserum against purified HetR or HetZ overproduced in *E*s*cherichia coli*, visualized using alkaline phosphatase-conjugated secondary antibody specific for rabbit IgG (Thermo Scientific, Waltham, USA) with NBT and BCIP as substrates. Two biological repeats showed similar results.

## Results

### Upregulated expression from P_*hetR*_ and P_*patS*_ in *hetR*-minus heterocysts

In a *hetR*-minus mutant, heterocyst differentiation is not initiated, and genes otherwise specifically expressed in heterocysts are mostly not upregulated after nitrogen stepdown. Such genes could be directly or indirectly regulated by HetR. Under our conditions, co-expression of *hetZ* and *hetP* from P_*ntcA*_ (rather than expression of *hetZ* or *hetP* alone from the same promoter) enabled the *hetR* mutant, 7120*hetR*::C.CE2, to form functional heterocysts at the ends of filaments [34]. Such a phenotype was probably due to the lack of expression of *patA*, a gene required for heterocyst formation at intercalary positions, in vegetative cells of the *hetR* mutant [31]. Formation of functional *hetR*-minus heterocysts [34] indicated that genes required for the function of heterocysts could be properly expressed without HetR, but it gave no information about the regulation of P_*hetR*_ and P_*patS*_ in (pro)heterocysts. Using *gfp* (green fluorescence protein) as the reporter gene, we tested the promoters of *hetR* and *patS* in (pro)heterocysts without HetR.

**Fig 1:**
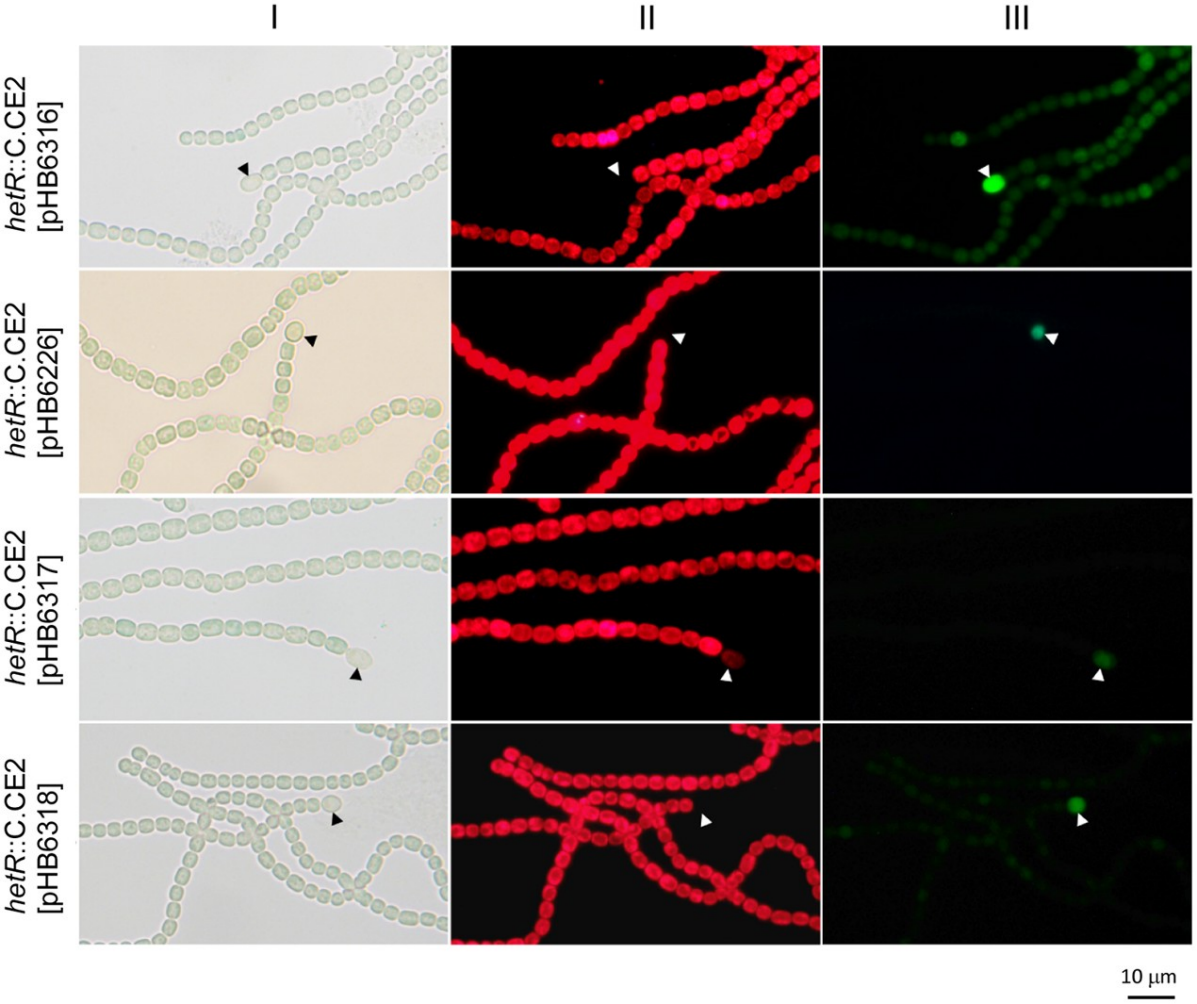
Light (I), autofluorescence (II) and GFP fluorescence (III) photomicrographs of *Anabaena* 7120 *hetR*::C.CE2 harboring plasmids with P_*ntcA*_-*hetP*-*hetZ* and Ω-promoter-*gfp*. On pHB6316, pHB6226, pHB6317 and pHB6318, *gfp* was expressed from P_*hetR*_, P_*patS*_, P_*hepB*_ and P_*hglD*_, respectively. Photomicrographs were taken at 24 h after nitrogen stepdown. Solid arrowheads point to heterocysts.

Plasmids carrying P_*ntcA*_-*hetZ*-*hetP* and the structure ‘Ω-promoter-*gfp*’ (the Ω cassette terminates background transcription, ref. [44]) were constructed and introduced into the *hetR* mutant. The tested promoters included P_*hetR*_, P_*patS*_, P_*hepB*_ and P_*hglD*_. *hepB* and *hglD* are genes involved in the formation of heterocyst envelope polysaccharide layer and glycolipid layer respectively, therefore P_*hepB*_ and P_*hglD*_ were included as the controls for heterocyst-specific expression [41]. Without a functional *hetR*, overexpression of *hetZ* and *hetP* led to heterocyst formation at the ends of filaments and upregulated expression of *gfp* from P_*hetR*_, P_*patS*_, P_*hepB*_ and P_*hglD*_ in heterocysts relative to that in vegetative cells (Fig 1). Clearly, HetR is not essential for the expression from all these promoters.

### Upregulation of *patS* in heterocysts depends on the DIF1 motif and *hetZ*

Like *hetR*, *hetZ* and *hetP*, *patS* is upregulated in *Anabaena* 7120 shortly after nitrogen stepdown (S1A Fig). In the *hetR* mutant, *patS* could be upregulated by overexpression of *hetZ* rather than *hetP* (S1B Fig). Consistently, *patS* was upregulated in a Δ*hetP* mutant but not in a Δ*hetZ* mutant [35] or a *hetZ*::Tn*5*-1087b mutant [32] of *Anabaena* 7120. These results implied that the upregulation of *patS* is dependent on HetZ rather than HetR.

**Fig 2:**
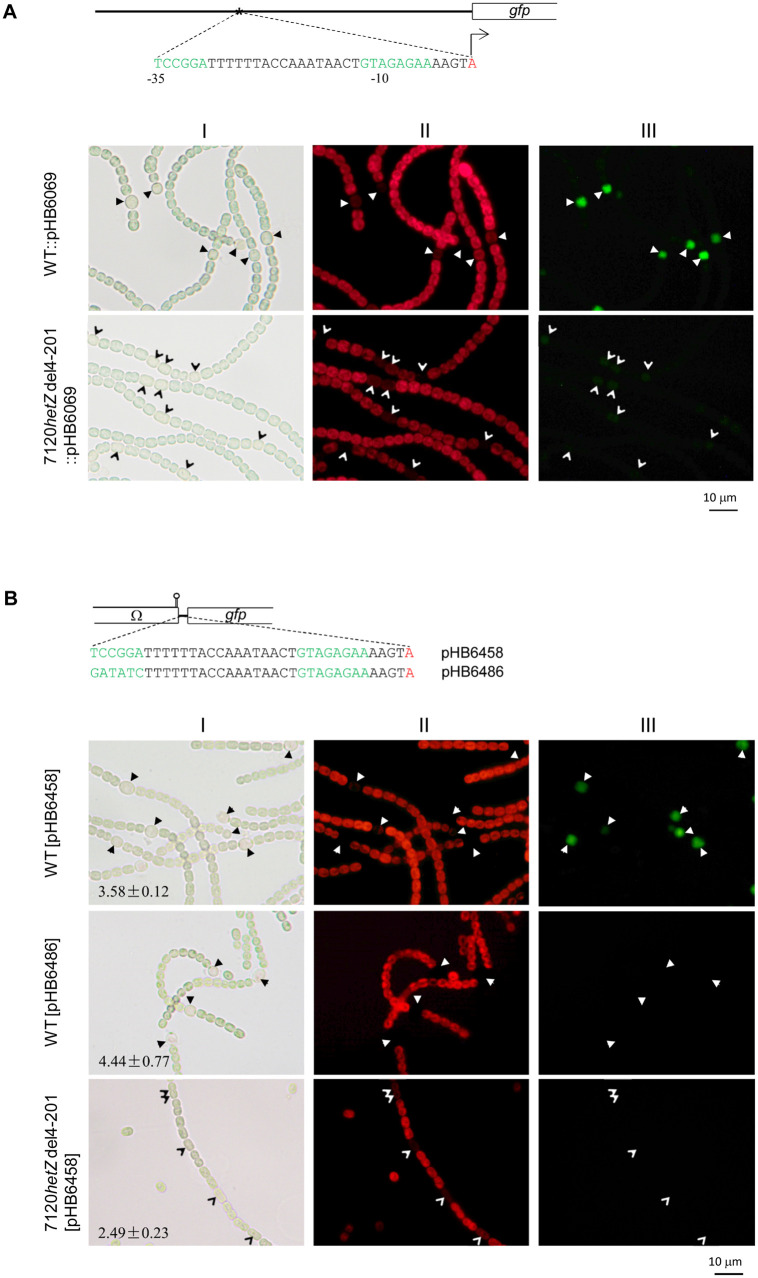
Light (I), autofluorescence (II) and GFP fluorescence (III) photomicrographs of *Anabaena* 7120 and 7120*hetZ* del4-201, with *gfp* expressed from the full-length promoter or DIF1-motif promoter of *patS*. Photomicrographs were taken at 24 h after nitrogen stepdown. Solid and empty arrowheads point to heterocysts and differentiating cells, respectively. Means ± SD are relative copy numbers of plasmids (relative to the copy number of *rnpB* in the genome). (A) Expression of *gfp* from the full-length *patS* promoter in the genome. The plasmid pHB6069 with P_*patS*_-*gfp* was integrated into the chromosome of *Anabaena* 7120 and the *hetZ* mutant via homologous single-crossover recombination. In the schematic diagram for the structure of full-length P_*patS*_ fused to *gfp*, the bent line with an empty arrowhead indicates the transcription start point of the DIF1-motif promoter. (B) Expression of *gfp* from the minimal DIF1-motif promoter on zeta-based plasmids in *Anabaena* 7120 and the *hetZ* mutant. pHB6486 and pHB6458 are plasmids with the minimal DIF1-motif promoter of *patS*, in which TCCGGA was substituted or not. The stem-loop structure stands for the transcription terminator at the end of Ω cassette.

To confirm the role of HetZ in expression of *patS*, we further generated a partial deletion mutant, 7120*hetZ*del4-201, of *Anabaena* 7120 with 66 amino acids near the N-terminus of HetZ deleted in frame while preserving the putative promoter internal to *hetZ* serving *patU5*-*patU3* [32]. This mutant showed no morphologically discernible heterocyst differentiation but formed some cells with less autofluorescence after nitrogen stepdown. These cells initiated differentiation, but the differentiation process ceased at the very early stage. The decreased autofluorescence was due to the degradation of phycobilisomes [45]. A non-replicative plasmid (pHB6069) containing P_*patS*_ (-1070 ~ +48 relative to the translational start site of *patS*) upstream of *gfp* was integrated into the genomes of *Anabaena* 7120 and the derivative strain 7120*hetZ*del4-201 via homologous single-crossover recombination. *Anabaena* 7120::pHB6069 showed moderate expression of *gfp* specifically in (pro)heterocysts, whereas 7120*hetZ*del4-201::pHB6069 showed much weaker (but visible) expression of *gfp* in differentiating cells (Fig 2A).

Employing *gfp* as a reporter gene in *Anabaena* 7120, we delimited the promoter of *patS* to the region -662 ~ -457 upstream of the start codon (S2 Fig, see photomicrographs for expression of *gfp* from fragments i, ii and iii). In this region, there is a DIF1 motif (TCCGGA) located 35 bp upstream of the tsp (transcriptional start point) -580 of *patS* [39]. We constructed a zeta-based plasmid with the minimal DIF1-motif promoter (a 41-bp fragment) positioned upstream of *gfp* (pHB6458) and a similar plasmid with TCCGGA replaced with GATATC (pHB6486). GFP was expressed in (pro)heterocysts of *Anabaena* 7120 [pHB6458] but not in differentiating cells of 7120*hetZ*del4-201 carrying the same plasmid; substitutions at TCCGGA abolished the expression of *gfp* in the wild-type strain (Fig 2B). These results established that activation of *patS* in (pro)heterocysts largely depends on HetZ and the DIF1-motif promoter. Similarly, expression from the DIF1-motif promoter of *patX* is also dependent on the function of *hetZ* (S3 Fig).

### Upregulation of *hetR* in heterocysts depends on the DIF1 motif and *hetZ*

As shown with RT-qPCR, *hetR* was upregulated in the *hetZ* mutant 7120*hetZ*del4-201 at 6 h after nitrogen stepdown (S4 Fig). However, the expression of *hetR* in *hetZ* mutants was probably not patterned [32].

**Fig 3:**
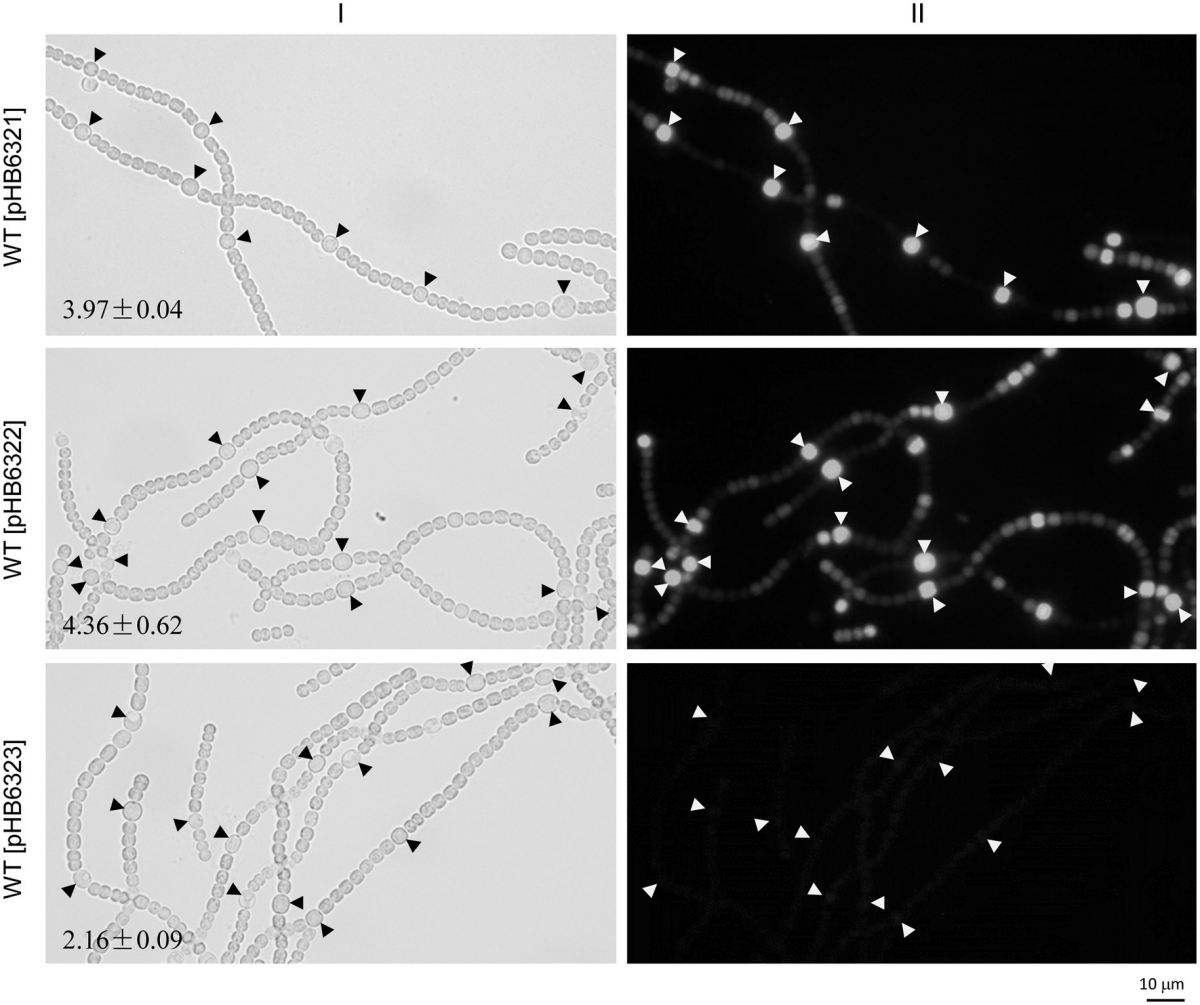
Light (I) and GFP fluorescence (II) photomicrographs of *Anabaena* 7120 derivatives with *gfp* expressed from the wild-type or mutated promoter of *hetR*. pHB6321: with the wild-type promoter (-695 ~ -250) of *hetR*; pHB6322: with GGGN_5_CCC (potential HetR-binding site) in the promoter of *hetR* substituted with AAAN_5_TTT; pHB6323: with TCCGGA (DIF1 motif) in the promoter of *hetR* substituted with CAATTG. Photomicrographs were taken at 24 h after nitrogen stepdown. Solid arrowheads point to heterocysts; means ± SD are relative copy numbers of plasmids.

*hetR* is an autoregulated gene [46], and a potential HetR-binding site has been identified upstream of the tsp -271 (for heterocyst-specific expression) [28, 31]. Upstream of the same tsp, there is also a potential DIF1-motif promoter [38]. To clarify the role of the HetR-binding site and the DIF1 motif in regulation of *hetR*, we compared the expression of *gfp* from the promoter (-695 ~ -250 relative to the translational start site) of *hetR* and the same DNA fragment without the HetR-binding site or the DIF1 motif. Expression from the promoter of *hetR* was upregulated in (pro)heterocysts of *Anabaena* 7120, and the upregulated expression was abolished by substitutions at the DIF1 motif but not at the HetR-binding site (Fig 3).

**Fig 4:**
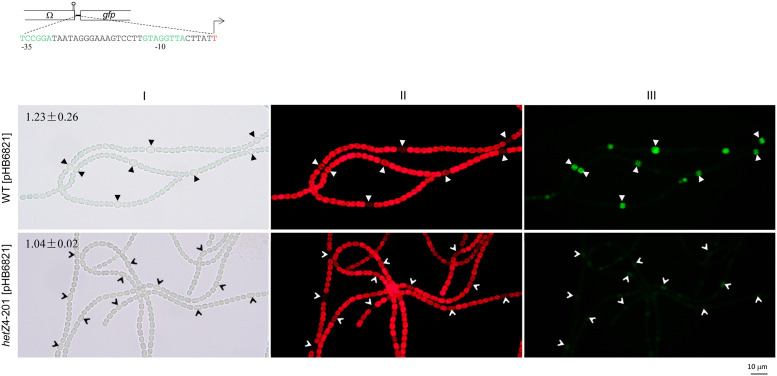
Expression of *gfp* from the minimal DIF1-motif promoter of *hetR* on a zeta-based plasmid in *Anabaena* 7120 and 7120*hetZ*del4-201. Top: the minimal sequence of DIF1-motif promoter cloned upstream of *gfp* in pHB6821. Photographs: light (I), autofluorescence (II) and GFP fluorescence (III) photomicrographs of *Anabaena* 7120 [pHB6821] and 7120*hetZ*del4-201 [pHB6821] at 24 h after nitrogen stepdown. *hetZ*4-201, 7120*hetZ*del4-201. Solid and empty arrowheads point to heterocysts and differentiating cells; relative copy numbers of plasmids are indicated as means ± SD.

To confirm the role of the DIF1 motif in heterocyst-specific expression of *hetR*, we constructed a zeta-based plasmid with the minimal DIF1-motif promoter (a 40-bp fragment) upstream of *gfp* (pHB6821) and introduced the plasmid into *Anabaena* 7120 and the *hetZ* mutant. As shown in Fig 4, GFP was expressed in (pro)heterocysts in *Anabaena* 7120 [pHB6821] but barely expressed in differentiating cells of the *hetZ* mutant. The copy numbers of zeta-based plasmids showed some changes in different strains but were still comparable with each other. Apparently, the upregulated expression of *hetR* in (pro)heterocysts is also mediated by HetZ via the DIF1 motif promoter.

**Fig 5:**
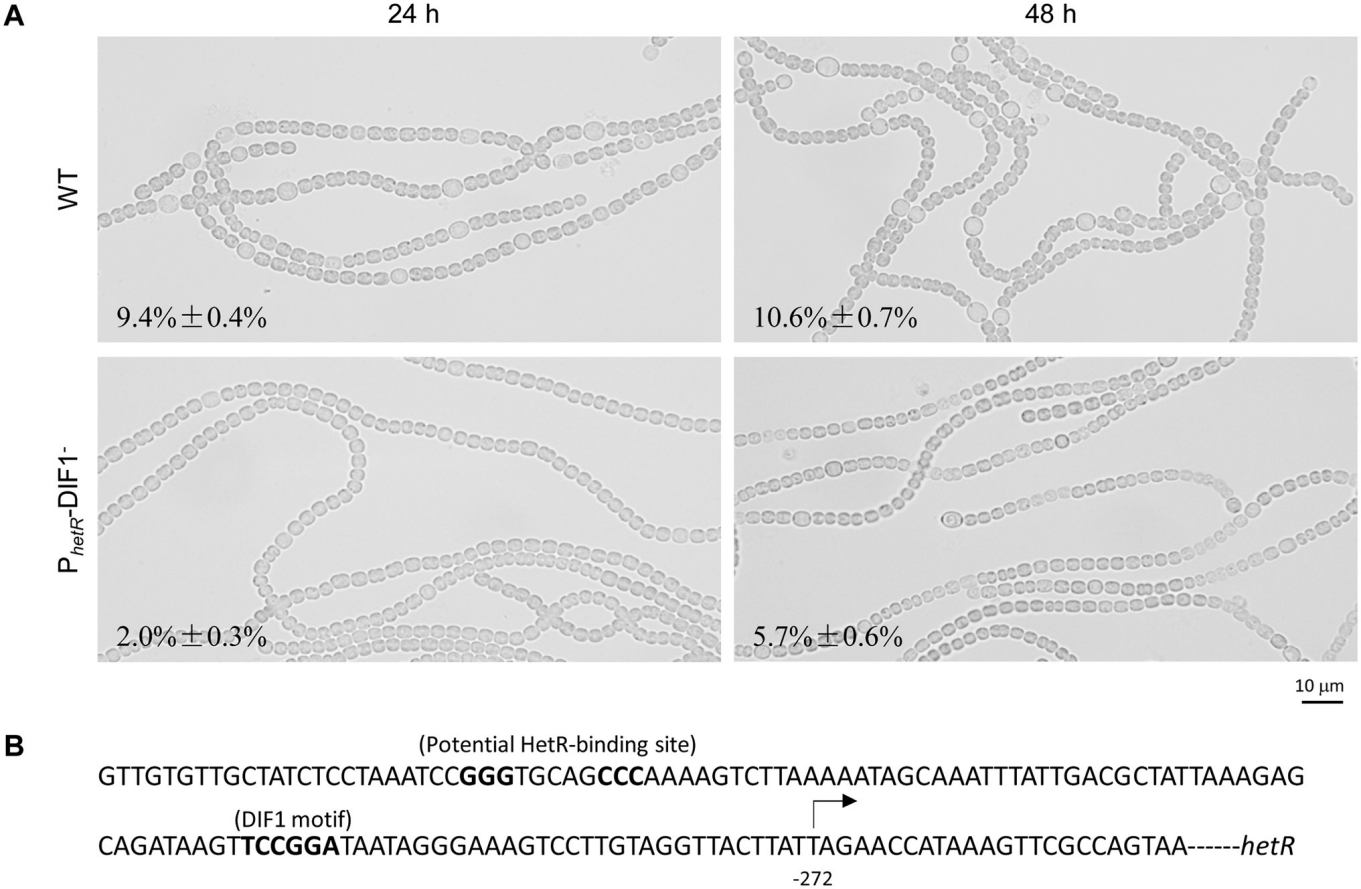
Differences between *Anabaena* 7120 and the P_*hetR*_-DIF1^-^ strain in heterocyst differentiation and expression of *hetR*. (A) Photomicrographs of *Anabaena* 7120 and the P_*hetR*_-DIF1^-^ strain at 24 h and 48 h after nitrogen stepdown. Frequencies of heterocysts/proheterocysts are indicated. (B) A stretch of sequence upstream of *hetR*, including the DIF1 motif, potential HetR-binding sequence and the tsp at -272.

We further generated a mutant of *Anabaena* 7120, P_*hetR*_-DIF1^-^, with the DIF1 motif substituted with GATATC in the chromosomal DNA. Compared to the wild type, the P_*hetR*_-DIF1^-^ strain showed delayed heterocyst differentiation and lowered heterocyst frequency (Fig 5). Using RACE-PCR, we confirmed that the tsp at nucleotide -272 (-271 in previous reports [16, 47]) upstream of *hetR* disappeared in P_*hetR*_-DIF1^-^. Clearly, the DIF1 motif is required for the heterocyst-specific expression of *hetR* and normal heterocyst differentiation.

### PatU3 interacts with HetZ and modulates the expression of *patS* and *hetR*

**Fig 6:**
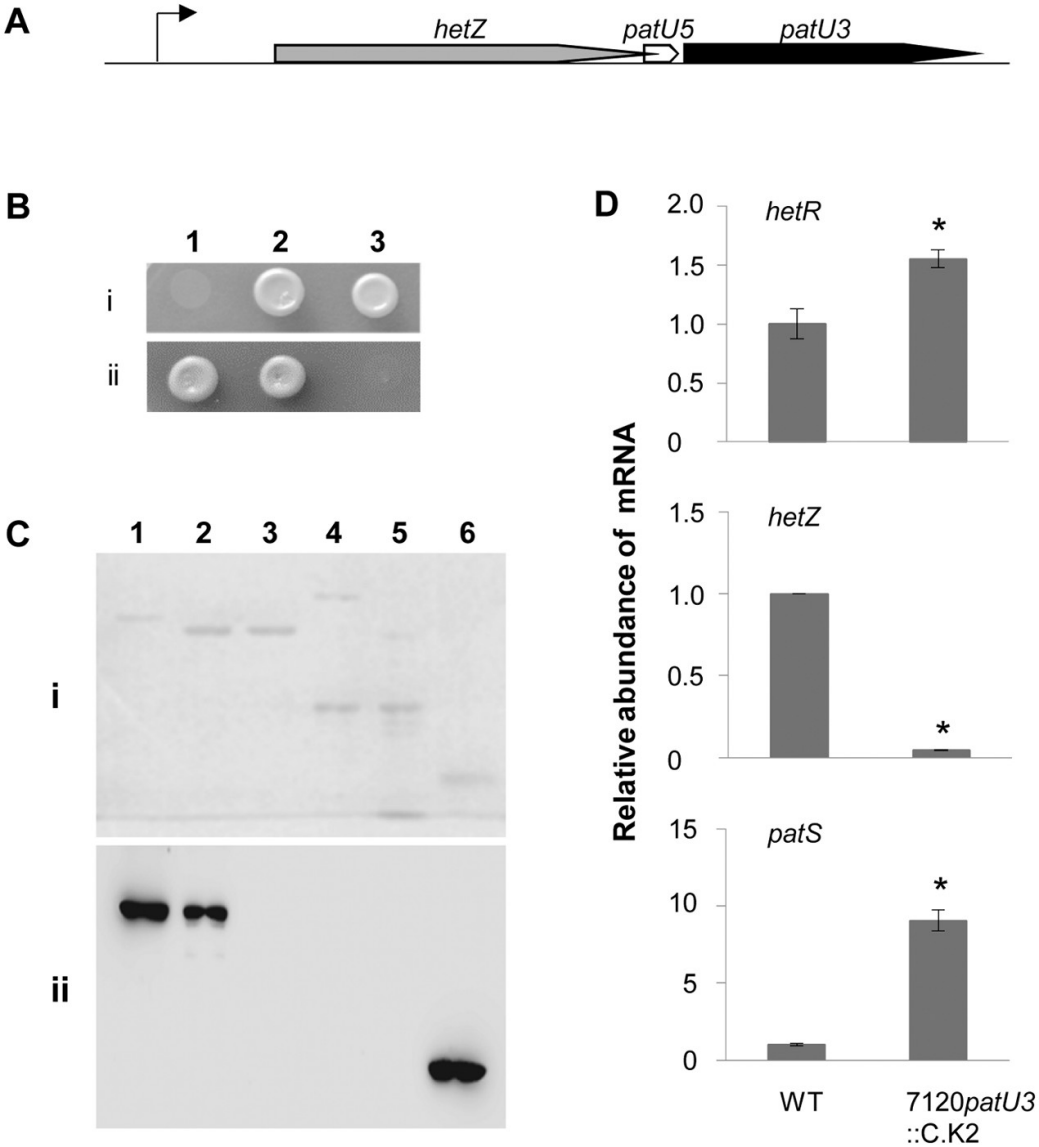
Interaction of PatU3 with HetZ. (A) The *hetZ*-*patU5*-*patU3* region. The bent line with an arrowhead indicates the tsp upstream of *hetZ*-*patU5*-*patU3*. Additional tsps within *hetZ* for *patU5*-*patU3* are not indicated. (B) Yeast two-hybrid assays of the interaction between PatU3 and HetZ. i) 1, pGBKT7-Lam + pGADT7-T, as the negative control; 2, pGBKT7-53 + pGADT7-T, as the positive control; 3, pGBKT7-PatU3 + pGADT7-HetZ. ii) 1, pGBKT7-PatU3 + pGADT7-HetZ[2–144]; 2, pGBKT7-PatU3 + pGADT7-HetZ[145–288]; 3, pGBKT7-PatU3 + pGADT7-HetZ[289–401]. Bracketed numbers (amino acid residue no.) indicate the portion deleted from HetZ (full length: 401 aa). (C) Pull-down assays of the interaction. Proteins were separated by SDS-PAGE (I) and analyzed with Western blot detection using anti-HA monoclonal antibody (II). 1, EF-Ts(HA)-HetZ; 2, MBP-PatU3 + MBP·Bind resin + EF-Ts(HA)-HetZ; 3, MBP-PatU3 + MBP·Bind resin + EF-Ts(HA); 4, MBP + MBP·Bind resin + EF-Ts(HA)-HetZ; 5, MBP + MBP·Bind resin + EF-Ts(HA); 6, EF-Ts(HA). (D) RT-qPCR analysis of mRNA abundance of *hetR*, *patS* and *hetZ* in *Anabaena* 7120 and the *patU3*::C.K4 mutant at 6 h after nitrogen stepdown. Data are means ± SD of 3 technical replicates. Asterisks indicate significant changes in mRNA abundance of *hetR*, *hetZ* and *patS* in the *patU3* mutant compared to that in the wild type.

*hetZ* and *patU3* play opposite roles in heterocyst differentiation, whereas *patU5* (which lies between *hetZ* and *patU3*) is not involved in heterocyst differentiation [32]. Employing the yeast two-hybrid system, we showed that PatU3 may interact with HetZ (Fig 6A-i); by a pull-down experiment, we confirmed the interaction between the two proteins (Fig 6B). As indicated in the two-hybrid assay, HetZ without the C-terminal portion no longer interacted with PatU3 (Fig 6A-ii).

The interaction between PatU3 and HetZ may modulate HetZ-dependent gene expression. Based on RT-qPCR analysis, we compared the expression of *hetR* and *patS* in the wild type and the 7120*patU3*::C.K4 strain at 6 h after nitrogen stepdown (Fig 6C). The mRNA level of *patS* was greatly increased in the *patU3* mutant relative to the wild type level, whereas that of *hetR* was slightly increased. Increased expression of *patS* probably inhibited the transcription of *hetZ* in the mutant (P_*hetZ*_-*gfp* in the mutant had shown a similar result, see ref. 32). However, the *patU3*::C.K4 mutation did not change the abundance of proteins HetR and HetZ in *Anabaena* filaments (S5 Fig).

## Discussion

HetR and PatS-derived peptides are key players for heterocyst differentiation and patterning in *Anabaena* 7120. How their encoding genes are regulated is an important question for understanding the molecular mechanism of the differentiation/patterning process. In this study, we showed that the DIF1 motif plays an important role in regulation of these genes and that the expression from DIF1 promoters depends on the function of *hetZ*.

HetR is often considered as the master regulator of heterocyst differentiation, and it directly regulates the expression of *hetP* [26] and *hetZ* [28] in developing heterocysts via HetR-recognition sequences and is required for the expression of *patA* in vegetative cells [31]. Whether HetR directly regulates the expression of *patS* and its own gene was a problem to be clarified. By examining gene expression in *hetR*-minus heterocysts, we were able to show that HetR is non-essential for the upregulated expression from promoters of *hetR* and *patS* during heterocyst differentiation. Therefore, HetR may control the expression of these genes through other regulatory factors, such as HetZ.

First, we showed that DIF1-motif promoters are responsible for the upregulation of *hetR*, *patS* and *patX* in (pro)heterocysts. Substitutions at the DIF1 motif greatly reduced the transcription activity of P_*patS*_; a mutant of *Anabaena* 7120 with the DIF1 motif of *hetR* substituted in the genome showed no transcription from the tsp -272 (or -271, the heterocyst-specific tsp in the wild type [47]). Upstream of *hetR*, there is also a potential HetR-recognition site, but that site was shown to be not required for the upregulated expression. Second, we showed that *hetZ* is required for the upregulated expression from these DIF1-motif promoters. *gfp* fused to minimal DIF1-motif promoters of *hetR*, *patS* and *patX* was specifically expressed in (pro)heterocysts in the wild type, and the expression was greatly weakened in the 7120*hetZ*del4-201 strain. These results indicated that HetZ directly or indirectly regulates the expression of these genes via DIF1 motif promoters.

For the results we presented, two points need to be addressed in particular. (1) How to explain the upregulation of P_*hetR*_ in a *hetR*-minus background? Because HetR and the global nitrogen regulator NtcA are dependent on each other for upregulated expression during heterocyst differentiation [48], lack of HetR would keep *ntcA* from being upregulated. For this question, we think that NtcA and HetR do not directly regulate each other’s encoding gene. In at least one substrain of *Anabaena* 7120, NrrA mediates the regulation of *hetR* by NtcA [49, 50]. Actually, formation of functional heterocysts in the *hetR* mutant with P_*ntcA*_-*hetZ*-*hetP* implied that genes regulated by NtcA were properly expressed in developing cells. Presumptively, the expression of *hetZ* and *hetP* from P_*ntcA*_ allowed sufficient expression of NtcA in those developing cells (the relationship between *hetZ*/*hetP* and *ntcA* awaits investigation), and NtcA in turn enhanced the expression of P_*ntcA*_-*hetZ*-*hetP* and indirectly upregulated P_*hetR*_. (2) How to explain the differentiating cells in the 7120del*hetZ*4-201 mutant? In the *hetZ* mutant generated with *Anabaena* 7120 in our laboratory (substrain IHB), we found that the mRNA level of *hetR* was increased after nitrogen stepdown as in the wild type (S4 Fig), therefore the expression of *hetR* could have initiated cell differentiation that ceased at the very early stage in less regular pattern (consistent with the low expression of *patS*). This is a difference between the *hetZ* mutant generated in this study and that reported by Videau et al [35].

**Fig 7:**
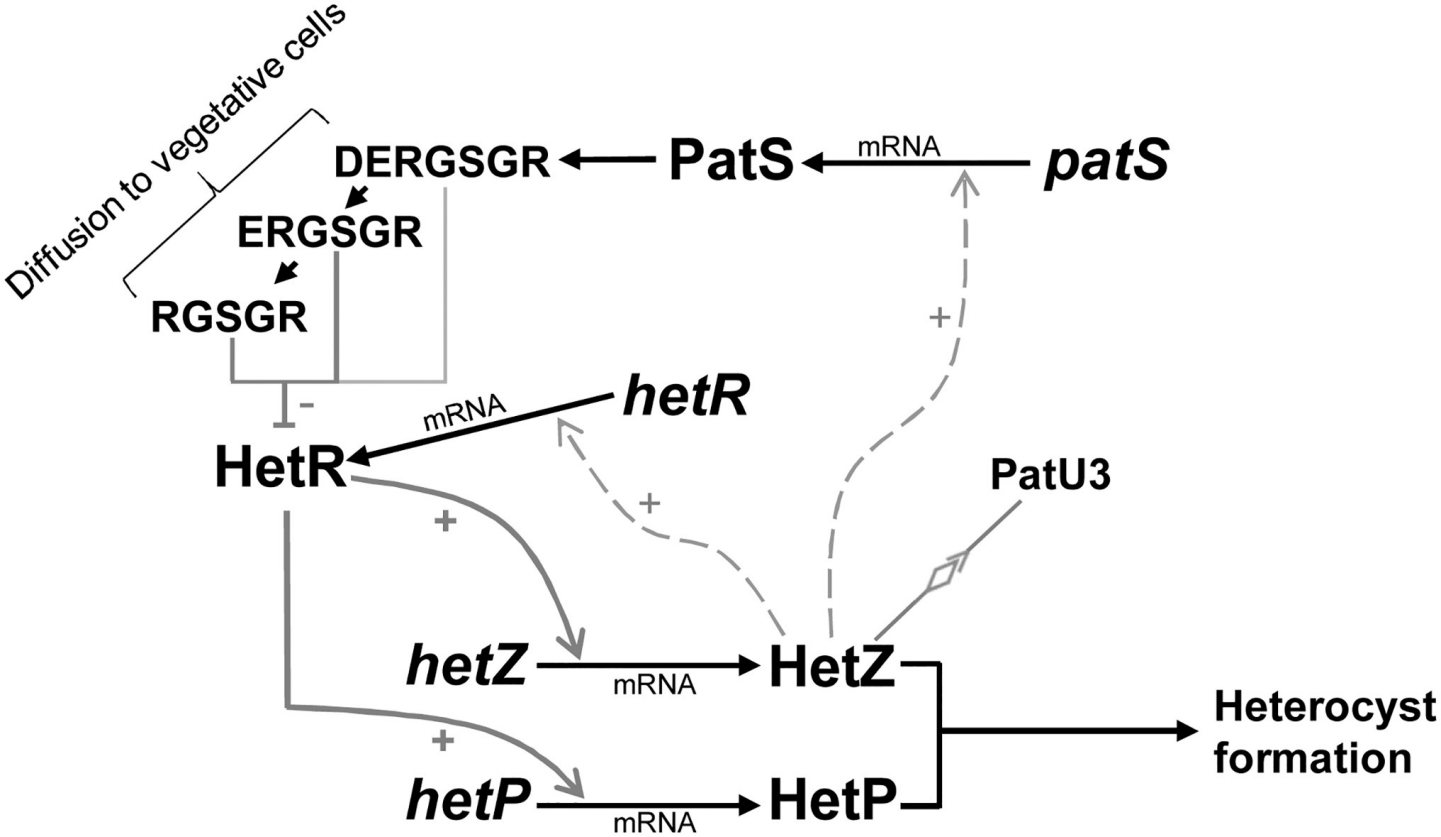
A schematic diagram showing the core regulatory circuit for heterocyst differentiation in *Anabaena* 7120. Dark lines with a solid arrowhead indicate gene expression, processing of peptide or cell differentiation; grey lines with an open arrowhead (+) or T-shaped end (-) indicate activation or inhibition of gene expression or protein activity (solid lines for confirmed direct interaction/regulation, dashed lines for direct or indirect regulation). The diamond-Y fork indicates protein-protein interaction. Thickness and darkness of lines roughly indicate the strength of interaction/regulation. Processing of PatS, regulation of *hetP* and regulation of *hetZ* are described in references 18, 26 and 28. HetN (for maintenance of heterocyst pattern), PatA (for heterocyst formation at intercalary positions), PatX (not required for heterocyst patterning in *Anabaena* 7120), and other factors that affect heterocyst differentiation/patterning, are not shown here.

As a gene directly regulated by HetR, *hetZ* is involved in initiation of heterocyst differentiation and regulation of *patS*/*patX* and *hetR*. *patX* is not required for de novo heterocyst patterning in *Anabaena* 7120 (Du Y, Gao H and Xu X, unpublished), but its counterparts in most other heterocyst-forming species may play a role in heterocyst patterning. Therefore, HetR, HetZ and PatS/PatX form the core regulatory circuit in most heterocyst-forming cyanobacteria. This conclusion is important, because HetZ may provide an additional site for modulation of the expression of *patS*/*patX*, the sources of diffusible inhibitors for de novo pattern formation. PatU3 is a candidate for the modulator. It interacts with HetZ and somehow modulates the expression of *hetR*, *hetZ* and *patS* (Fig 6). Presumptively, interaction with PatU3 can regulate the cellular concentration of free HetZ therefore modulate HetZ-dependent gene expression. Alternatively, PatU3 may have additional functions that indirectly affect the expression of these genes. The core regulatory circuit of heterocyst differentiation in *Anabaena* 7120 is summarized in Fig 7. This coordination scenario involving multiple activating/inhibiting factors may help to refine the current models [51, 52] for heterocyst differentiation and patterning.

## Supporting information

S1 Fig RT-qPCR analyses showing the upregulation of *patS* in *Anabaena* 7120 and the relationship between the expression *hetZ* and *patS* in a *hetR*-minus background. (PDF)

S2 Fig Expression of *gfp* from fragments upstream of *patS* on a pDU1-based plasmid in *Anabaena* 7120. (PDF)

S3 Fig Light (I), autofluorescence (II) and GFP fluorescence (III) photomicrographs showing the expression of *gfp* from the DIF1-motif promoter of P_*patX*_ in *Anabaena* 7120 and 7120*hetZ*del4-201. (PDF)

S4 Fig RT-qPCR analysis of the expression of *patS* and *hetR* in the wild type and the mutant 7120*hetZ*del4-201 at 0 and 6 h after nitrogen stepdown. (PDF)

S5 Fig Western blot detection of HetR and HetZ in the wild type and the *patU3* mutant of *Anabaena* 7120 at 24 h after nitrogen stepdown. (PDF)

S1 Table *Anabaena* strains, plasmids and primers. (PDF)

S1 Raw images (PDF)
