# Correction: Expression from DIF1-motif promoters of *hetR* and *patS* is dependent on HetZ and modulated by PatU3 during heterocyst differentiation

**DOI:** 10.1371/journal.pone.0269050

**Published:** 2022-05-23

**Authors:** Yaru Du, He Zhang, Hong Wang, Shuai Wang, Qiqin Lei, Chao Li, Renqiu Kong, Xudong Xu

In Fig 5, the bent arrow, which indicates the transcription start point, and ‘-272’ are positioned incorrectly in the sequence. The electrophoretogram of the 5′ RACE-PCR products of *hetR* transcripts in the wild type and the mutant is missing. Please see the correct Fig 5 here.

**Fig 5:**
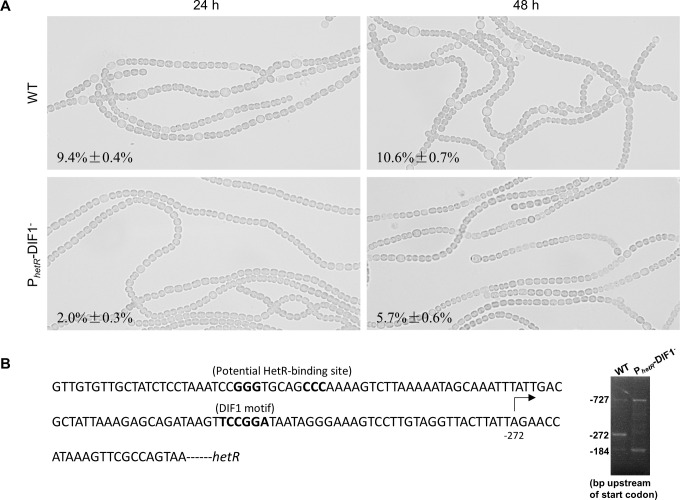
Differences between *Anabaena* 7120 and the P_*hetR*_-DIF1^-^ strain in heterocyst differentiation and expression of *hetR*. (A) Photomicrographs of *Anabaena* 7120 and the P_*hetR*_-DIF1^-^ strain at 24 h and 48 h after nitrogen stepdown. Frequencies of heterocysts/proheterocysts are indicated. (B) A stretch of sequence upstream of *hetR*, including the DIF1 motif, potential HetR-binding sequence and the tsp at -272 (left), and the electrophoretogram of the 5′ RACE-PCR products of *hetR* transcripts in the two strains at 18 h after nitrogen stepdown (right). Each band of the RACE-PCR (indicated with tsp) was confirmed by sequencing.

Accordingly, the caption for Fig 5 has been updated to include updated information. Please see the complete, correct Fig 5 caption here.

In the Discussion, there is an error in the first two sentences of the third paragraph. The correct sentences are:

First, we showed that the DIF1-motif promoter is responsible for the upregulation of *hetR* in (pro)heterocysts. Substitutions at the DIF1 motif greatly reduced the transcription activity of P_*hetR*_; a mutant of *Anabaena* 7120 with the DIF1 motif of *hetR* substituted in the genome showed no transcription from the tsp -272 (previously -271, the heterocyst-specific tsp in the wild type [47]).
