# Supplementary material for: Expression from DIF1-motif promoters of hetR and patS is dependent on HetZ and modulated by PatU3 during heterocyst differentiation
Source: PLoS One. 2020 Jul 23;15(7):e0232383. doi: 10.1371/journal.pone.0232383 (PMC7377430; doi:10.1371/journal.pone.0232383)
Supplement: S1 Fig — (PDF) [file pone.0232383.s001.pdf]

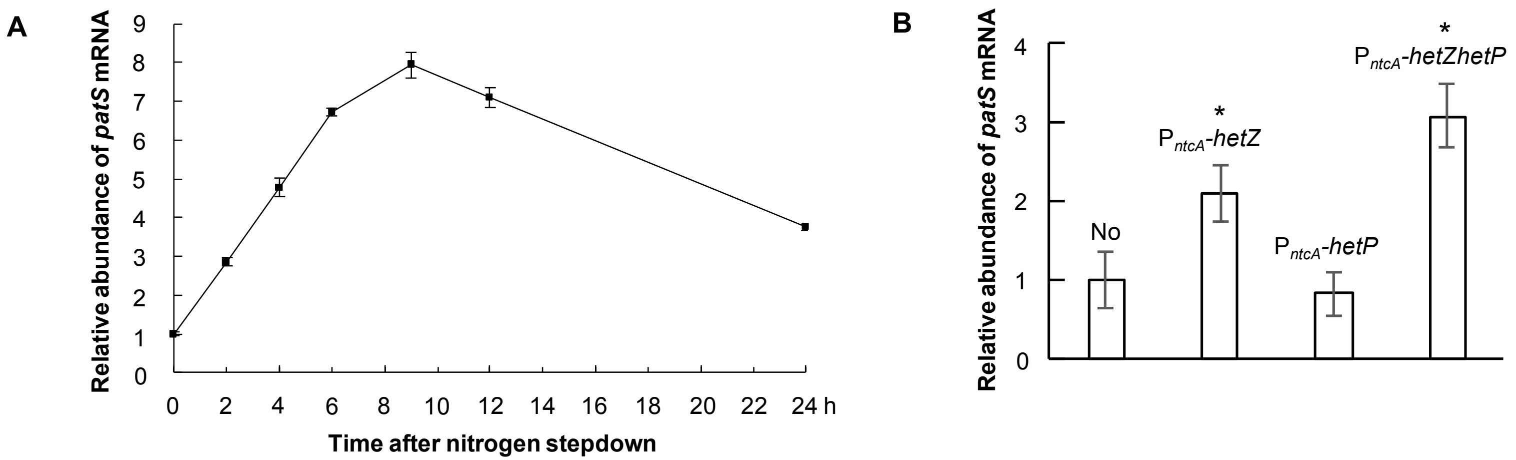

S1 Fig. RT-qPCR analyses showing the upregulation of *patS* in *Anabaena* 7120 after nitrogen stepdown and the relationship between the expression of *hetZ* and *patS* in a *hetR*-minus background. (A) Induced expression of *patS* after nitrogen stepdown. The RT-qPCR was performed with the same mRNA samples as previously used for analyses of transcription of *hetR*, *hetP* and *hetZ* (see Fig.S1 of Zhang et al, 2018), using a pair of primers located at -35~-14 bp (patS-RT-1) and 29~50 (patS-RT-2) (see Table S1) . Under our conditions, all these genes were upregulated at 2 h after nitrogen stepdown. (B) Relative abundance of *patS* mRNA in the *hetR* ::C.CE2 mutant expressing *hetZ*, *hetP* or both *hetZ* and *hetP* at 6 h after nitrogen stepdown. No, neither *hetZ* nor *hetP* was overexpressed in the *hetR* mutant. Apparently, the expression of *patS* was dependent on *hetZ* rather than *hetP*. Data are means ± SD of 3 biological replicates; asterisks indicate significantly higher expression of *patS* (p-value < 0.05) compared to that in the *hetR* mutant.

Zhang H, Wang S, Wang Y, Xu X. Functional overlap of *hetP* and *hetZ* in regulation of heterocyst differentiation in *Anabaena* sp. strain PCC 7120. J Bacteriol. 2018; 200: e00707-17.
