# Supplementary material for: Expression from DIF1-motif promoters of hetR and patS is dependent on HetZ and modulated by PatU3 during heterocyst differentiation
Source: PLoS One. 2020 Jul 23;15(7):e0232383. doi: 10.1371/journal.pone.0232383 (PMC7377430; doi:10.1371/journal.pone.0232383)
Supplement: S2 Fig — (PDF) [file pone.0232383.s002.pdf]

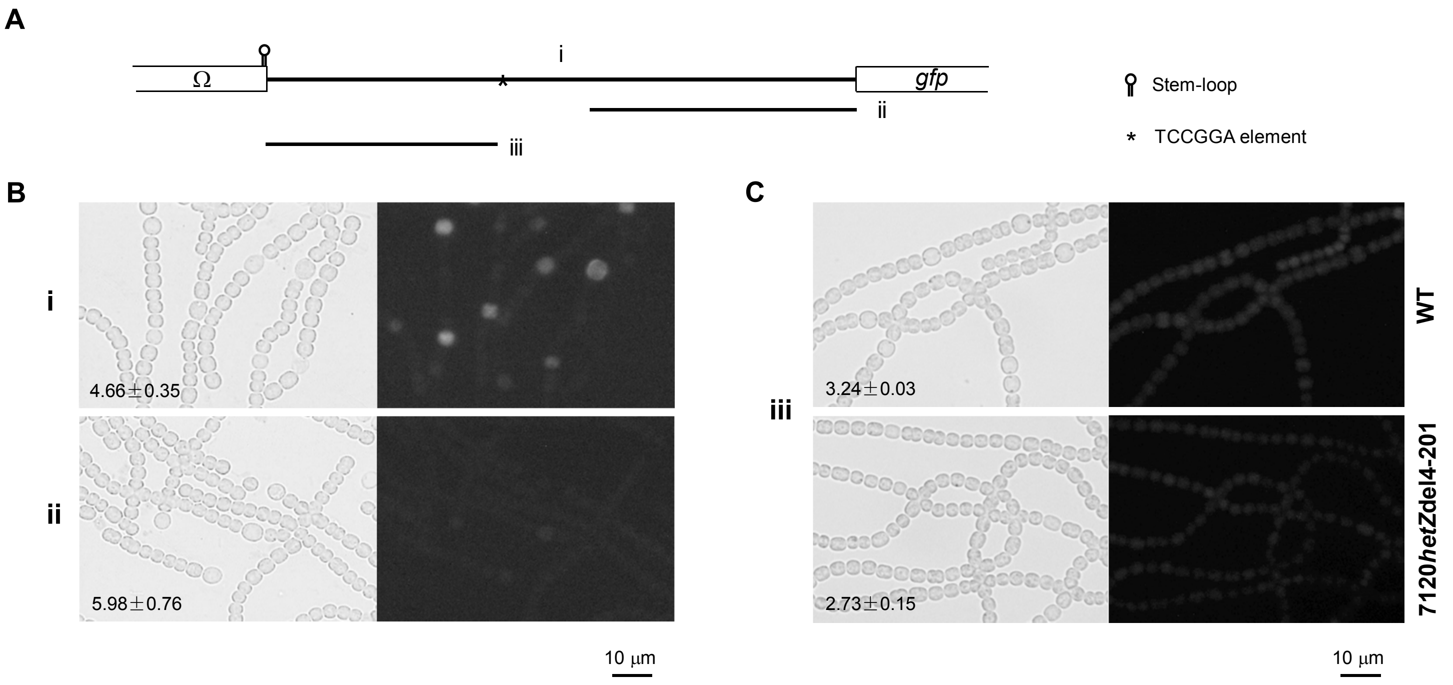

S2 Fig. Expression of *gfp* from fragments upstream of *patS* on a pDU1-based plasmid in *Anabaena* 7120 and the *hetZ* mutant. Relative copy numbers of plasmids are indicated as means±SD. (A) Fragments upstream of *patS* (see Table S1 for details). i: -1070 ~ +48, pHB1125; ii: -457 ~ +46, pHB5757; iii: -1070 ~ -662, pHB6668. (B) Light (left) and GFP fluorescence (right) photomicrographs of *Anabaena* 7120 harboring plasmids with *gfp* fused to fragment i or ii upstream of *patS* at 24 h after nitrogen stepdown. (C) Light (left) and GFP fluorescence (right) photomicrographs showing the expression of *gfp* from fragment iii in the wild type (WT) and the *hetZ* mutant of *Anabaena* 7120 at 24 h after nitrogen stepdown.
