# Supplementary material for: Expression from DIF1-motif promoters of hetR and patS is dependent on HetZ and modulated by PatU3 during heterocyst differentiation
Source: PLoS One. 2020 Jul 23;15(7):e0232383. doi: 10.1371/journal.pone.0232383 (PMC7377430; doi:10.1371/journal.pone.0232383)
Supplement: S3 Fig — (PDF) [file pone.0232383.s003.pdf]

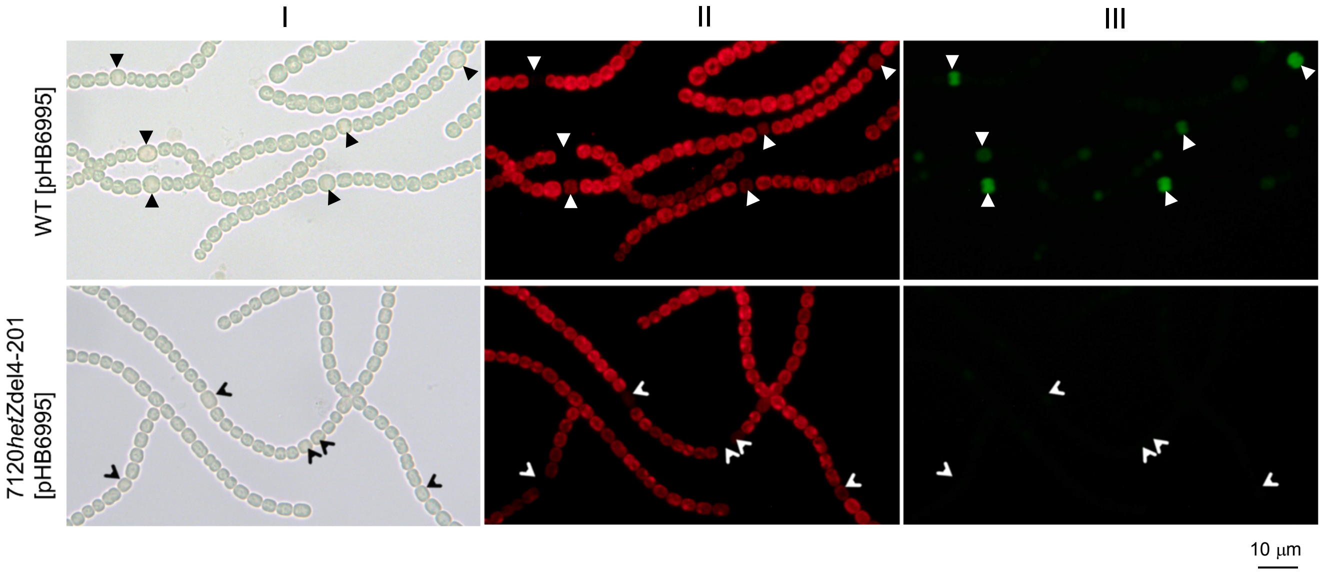

S3 Fig. Light (I), autofluorescence (II) and GFP fluorescence (III) photomicrographs showing the expression of *gfp* from the DIF1-motif promoter of P_{patX} in *Anabaena* 7120 [pHB6995] and 7120*hetZ*del4-201 [pHB6995]. WT, wild type. pHB6995, a plasmid with the minimal DIF1-motif promoter of P_{patX} cloned upstream of *gfp* (see Table S1). Solid arrows point to heterocysts, empty arrows point to differentiating cells in the *hetZ* mutant.
