# Supplementary material for: Expression from DIF1-motif promoters of hetR and patS is dependent on HetZ and modulated by PatU3 during heterocyst differentiation
Source: PLoS One. 2020 Jul 23;15(7):e0232383. doi: 10.1371/journal.pone.0232383 (PMC7377430; doi:10.1371/journal.pone.0232383)
Supplement: S4 Fig — (PDF) [file pone.0232383.s004.pdf]

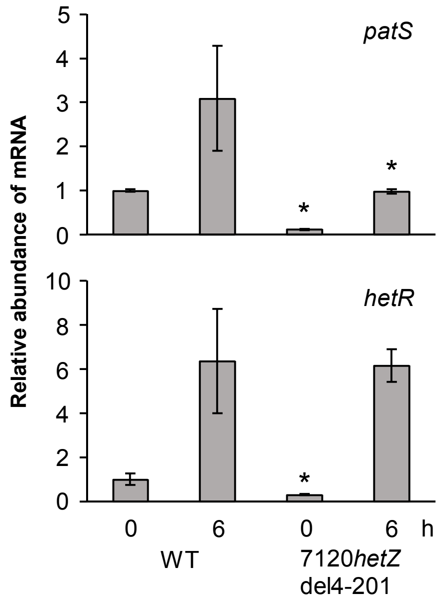

S4 Fig. RT-qPCR analysis of the expression of *patS* and *hetR* in the wild type and *hetZ* mutant of *Anabaena* 7120 at 0 and 6 h after nitrogen stepdown. Asterisks indicate significantly lower expression of *patS* or *hetR* (p-value < 0.05) in the *hetZ* mutant relative to the wild type level.
