# Supplementary material for: Expression from DIF1-motif promoters of hetR and patS is dependent on HetZ and modulated by PatU3 during heterocyst differentiation
Source: PLoS One. 2020 Jul 23;15(7):e0232383. doi: 10.1371/journal.pone.0232383 (PMC7377430; doi:10.1371/journal.pone.0232383)
Supplement: S5 Fig — (PDF) [file pone.0232383.s005.pdf]

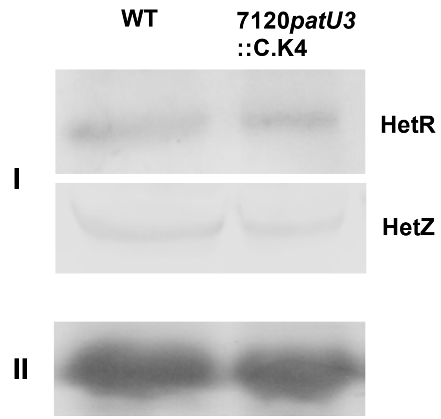

S5 Fig. Western blot detection of HetR and HetZ in the wild type and the *patU3* mutant of *Anabaena* 7120 at 24 h after nitrogen stepdown. (I) Western blots; (II) a part of CBB-stained SDS-PAGE gel image, showing that equal amounts of proteins were loaded.
