# Supplementary material for: Expression from DIF1-motif promoters of hetR and patS is dependent on HetZ and modulated by PatU3 during heterocyst differentiation
Source: PLoS One. 2020 Jul 23;15(7):e0232383. doi: 10.1371/journal.pone.0232383 (PMC7377430; doi:10.1371/journal.pone.0232383)
Supplement: S1 Table — (PDF) [file pone.0232383.s006.pdf]

S1 Table. *Anabaena* strains, plasmids and primers

**Strains**	**Derivation and relevant characteristics** [a]	**Reference or source**
WT	wild type of *Anabaena* sp. PCC 7120	FACHB [b]
WT::pHB6069	Sm^rSp^r, pHB6069 with *gfp* fused to the full-length promoter (-1070 ~ +48) of *patS*, integrated into the genome of *Anabaena* 7120 via single crossover	This study
WT [pHB6321]	Sm^rSp^r, pHB6321 with the promoter (-695 ~ -250) of *hetR* upstream of *gfp* introduced into the wild type	
WT [pHB6322]	Sm^rSp^r, pHB6322, similar to pHB6321 but with GGGN_5CCC (HetR-binding site) in the *hetR* promoter substituted with AAAN_5TTT, introduced into the wild type	
WT [pHB6323]	Sm^rSp^r, pHB6323, similar to pHB6321 but with TCCGGA (DIF^+ motif) in the *hetR* promoter substituted with CAATTG, introduced into the wild type	
WT [pHB6458]	Sm^rSp^r, pHB6458 with the minimal DIF^+ motif promoter of *patS* upstream of *gfp* introduced into the wild type	
WT [pHB6486]	Sm^rSp^r, pHB6486 with the mutated minimal DIF^+ motif promoter (TCCGGA substituted with GATATC) of *patS* upstream of *gfp* introduced into the wild type	
WT [pHB6821]	Sm^rSp^r, pHB6821 with the minimal DIF^+ motif promoter of *hetR* upstream of *gfp* introduced into the wild type	
7120*hetR*::C.CE2	Cm^rEm^r, *hetR* mutant, with C.CE2 inserted into the ClaI site of *hetR*	Du et al, 2012
7120*hetR*::C.CE2 [pHB6226]	$Cm^rEm^rSm^rSp^r$, pHB6226 carrying Ω-P_{patS}-*gfp* and P_{ntcA}-*hetZ*-*hetP* introduced into 7120*hetR*::C.CE2	This study
7120*hetR*::C.CE2 [pHB6316]	$Cm^rEm^rSm^rSp^r$, pHB6226 carrying Ω-P_{hetR}-*gfp* and P_{ntcA}-*hetZ*-*hetP* introduced into 7120*hetR*::C.CE2	
7120*hetR*::C.CE2 [pHB6317]	$Cm^rEm^rSm^rSp^r$, pHB6226 carrying Ω-P_{hepB}-*gfp* and P_{ntcA}-*hetZ*-*hetP* introduced into 7120*hetR*::C.CE2	
7120*hetR*::C.CE2 [pHB6318]	$Cm^rEm^rSm^rSp^r$, pHB6226 carrying Ω-P_{hglD}-*gfp* and P_{ntcA}-*hetZ*-*hetP* introduced into 7120*hetR*::C.CE2	
7120*het*Zdel4-201	*hetZ* in-frame deletion mutant, with bp 4-201 deleted, generated by homologous double crossover between pHB5553 and the chromosome of *Anabaena* sp. PCC 7120	
7120*het*Zdel4-201 ::pHB6069	Sm^rSp^r, pHB6069 with the full-length promoter (-1070 ~ +48) of *patS* upstream of *gfp*, integrated into the genome of 7120*het*Zdel4-201 via homologous single crossover	

	recombination	
7120*het*Zdel4-201 [pHB6458]	Sm^{r}Sp^{r}, pHB6458 with the minimal DIF^{+} motif promoter of *patS* upstream of *gfp* introduced into the *het*Zdel4-201 mutant	
7120*het*Zdel4-201 [pHB6821]	Sm^{r}Sp^{r}, pHB6821 with the minimal DIF^{+} motif promoter of *hetR* upstream of *gfp* introduced into the *hetZ* mutant	
P$_{hetR}$-DIF1^{-}	P$_{hetR}$ mutant, with TCCGGA upstream of *hetR* substituted with GATATC, generated by homologous double crossover between pHB6448 and the chromosome of *Anabaena* 7120	
**Plasmids**	**Derivation and relevant characteristics**	**Reference or source**
pACYC184	Cm^{r}Tc^{r}, a cloning vector with p15A origin of replication	Rose, 1988
pET28a-MBP	Km^{r}, maltose-binding protein gene cloned into pET28a	Haixia Xie, IHB, CAS
pET41a	Km^{r}, expression vector	Novagen
pGADT7	Ap^{r}, the prey vector	Clontech
pGADT7-T	Ap^{r}, T-antigen gene cloned in pGADT7	
pGBKT7	Km^{r}, the bait vector	
pGBKT7-53	Km^{r}, p53 gene cloned in pGBKT7	
pGBKT7-Lam	Km^{r}, Lamin C gene cloned in pGBKT7	
pHB247	Cm^{r}(Em^{r}), zeta plasmid with Tn*5*-1087b inserted between bp 2566 and 2567 (1 bp downstream of *asr9503*), retrieved from a Tn*5*-1087b generated mutant of *Anabaena* 7120	Ning and Xu, unpublished
pHB828	Ap^{r}Sm^{r}/Sp^{r}, Ω-*gfp* cloned in pMD18-T	Wang et al, 2005
pHB1071	Ap^{r}Sm^{r}/Sp^{r}, pUC19 ligated to pHB912, to provide multiple cloning sites upstream of the *gfp* reporter gene in the *E. coli*-*Anabaena* shuttle vector	
pHB1121	Sm^{r}/Sp^{r}, P$_{hepB}$ (-1199 ~ +51 relative to the start codon of *hepB*) cloned upstream of *gfp* in pHB1071, replacing the pUC19 portion	
pHB1123	Sm^{r}/Sp^{r}, P$_{hetR}$ (-1139 ~ +99 relative to the start codon of *hetR*) cloned upstream of *gfp* in pHB1071, replacing the pUC19 portion	
pHB1125	Sm^{r}/Sp^{r}, P$_{patS}$ (-1070 ~ +48 relative to the start codon of *patS*) cloned upstream of *gfp* in pHB1071, replacing the pUC19 portion	
pHB1128	Sm^{r}/Sp^{r}, P$_{hglD}$ (-603 ~ +84 relative to the start codon of *hglD*)	

	cloned upstream of *gfp* in pHB1071, replacing the pUC19 portion	
pHB3882	Ap^r, the PCR fragment (*Anabaena* sp. chromosomal bp 104854-105624) containing the *patU3* coding region amplified using primers patU3(MBP)-1 and patU3(MBP)-2, cloned into pMD18-T	This study
pHB3883	Km^r, *patU3* coding sequence excised with BamHI/XhoI from pHB3882, cloned into BamHI/XhoI-cut pET28a-MBP, to express MBP-PatU3 fusion protein	
pHB4197	Ap^r, the PCR fragment (*Anabaena* sp. chromosomal bp 3431199-3432348) containing *patU3* generated by PCR using primers alr0101-F/2-patU3-R, cloned into pMD18-T	
pHB4226	Ap^r, *hetZ* coding sequence cloned into pMD18-T	Zhang et al, 2018
pHB4344	Km^r, *patU3* excised with NdeI/EcoRI from pHB4197, cloned into NdeI/EcoRI-cut pGBKT7	This study
pHB4349	Ap^r, *hetZ* excised with NdeI/EcoRI from pHB4226, cloned into NdeI/EcoRI-cut pGADT7	
pHB4376	Km^r, *hetZ* coding sequence cloned into pET41a	Zhang et al, 2018
pHB4979	Ap^r, *E. coli tsf* (EF-Ts) with HA coding sequence generated by PCR using primers HA-tsf-F/HA-tsf-R, cloned into pMD18-T	This study
pHB5043	Km^r, EF-Ts(HA) coding sequence excised with BamHI/NdeI from pHB4979, cloned into BamHI/NdeI-cut pET-41a	
pHB5086	Km^r, *hetZ* coding sequence excised with BamHI/XhoI from pHB4376, cloned into BamHI/XhoI-cut pHB5043, to express EF-Ts(HA)-HetZ fusion protein	
pHB5328	Ap^r, *hetZ* lack of bp 4-432 bp generated by PCR using primers hetZ Mutant-AF/Mut-hetZ-R, cloned into pMD18-T	
pHB5329	Ap^r, *hetZ* lack of bp 433-864 generated by overlap PCR (Horton et al, 1989) using primer pairs Mut-hetZ-F/hetZ Mutant-BR and hetZ Mutant-BF/Mut-hetZ–R, cloned into pMD18-T	
pHB5330	Ap^r, *hetZ* lack of its 3′ end 342 bp generated by PCR using primers Mut-hetZ-F/hetZ Mutant-CR, cloned into pMD18-T	
pHB5346	Ap^r, *hetZ* lack of bp 4-432 bp excised with NdeI/XhoI from pHB5328, cloned into NdeI/XhoI-cut pGADT7	

pHB5347	Ap^{r}, *hetZ* lack of bp 433-864 excised with NdeI/XhoI from pHB5329, cloned into NdeI/XhoI-cut pGADT7	
pHB5348	Ap^{r}, *hetZ* lack of its 3′ end 342 bp excised with NdeI/XhoI from pHB5330, cloned into NdeI/XhoI-cut pGADT7	
pHB5552	Ap^{r}, a DNA fragment containing *hetZ* without bp 4-201 (*Anabaena* sp. chromosomal bp 102710-105007), generated by overlap PCR (Horton et al, 1989) using HetZ-up748-F/HetZ-201-1-R and HetZ-201-1-F/ HetZ-down344-R as the primer pairs, cloned into pMD18-T	This study
pHB5553	Sm^{r}Sp^{r}, the DNA fragment excised with SacI/XhoI from pHB5552, cloned into SacI/XhoI-cut pRL277, used to generate the *hetZ* [4-201] mutant of *Anabaena* 7120	
pHB6069	Sm^{r}Sp^{r}, pHB1125 cut with KpnI and NotI, blunted with T4 DNA polymerase and re-circularized with T4 DNA ligase	
pHB6206	Ap^{r}, a plasmid similar to pHB4907 described by Zhang et al (2018), with P$_{ntcA}$-*hetZ-hetP* generated by overlap PCR, cloned into pMD18-T	
pHB6226	Sm^{r}Sp^{r}, P$_{ntcA}$-*hetZ-hetP* excised with PvuII from pHB6206, cloned into KpnI-cut and T4 DNA polymerase-blunted pHB1125 (P$_{patS}$-*gfp*)	
pHB6310	Ap^{r}, a DNA fragment located -695 ~ -250 relative to the start codon of *hetR*, generated by PCR using primers PhetR p-1/p-2, cloned into pMD18-T	
pHB6311	Ap^{r}, a DNA fragment located -695 ~ -250 relative to the start codon of *hetR*, with GGGN$_5$CCC substituted with AAA N$_5$TTT, generated by overlap PCR using primers PhetR p-1/p-3 and PhetR p-4/p-2, cloned into pMD18-T	
pHB6312	Ap^{r}, a DNA fragment located -695 ~ -250 relative to the start codon of *hetR*, with TCCGGA substituted with CAATTG, generated by PCR using primers PhetR p-1/p-5, cloned into pMD18-T	
pHB6316	Sm^{r}Sp^{r}, P$_{ntcA}$-*hetZ-hetP* excised with PvuII from pHB6206, cloned into KpnI-cut and T4 DNA polymerase-blunted pHB1123 (P$_{hetR}$-*gfp*)	
pHB6317	Sm^{r}Sp^{r}, P$_{ntcA}$-*hetZ-hetP* excised with PvuII from pHB6206, cloned into KpnI-cut and T4 DNA polymerase-blunted pHB1121 (P$_{hepB}$-*gfp*)	
pHB6318	Sm^{r}Sp^{r}, P$_{ntcA}$-*hetZ-hetP* excised with PvuII from pHB6206,	

	cloned into KpnI-cut and T4 DNA polymerase-blunted pHB1128 (P_{hglD}-*gfp*)	
pHB6321	$Sm^r Sp^r$, P_{hetR} excised with SmaI and Sse8387I from pHB6310, cloned upstream of *gfp* in pHB1071, replacing the pUC19 portion	
pHB6322	$Sm^r Sp^r$, P_{hetR} with $GGGN_5CCC$ substituted with $AAAN_5TTT$, excised with SmaI and Sse8387I from pHB6311, cloned upstream of *gfp* in pHB1071, replacing the pUC19 portion	
pHB6323	$Sm^r Sp^r$, P_{hetR} with TCCGGA substituted with CAATTG, excised with SmaI and Sse8387I from pHB6312, cloned upstream of *gfp* in pHB1071, replacing the pUC19 portion	
pHB6368	Ap^r, a DNA fragment containing the encoding region of Em^r gene in pRL1087b (Ernst et al, 1992), generated with PCR using primers Em-1 and Em-2, cloned into pMD18-T	
pHB6379	$Ap^r Sm^r/Sp^r$, Ω-*gfp* excised with PstI (blunted with T4 DNA polymerase) and EcoRI from pHB828, cloned into BamHI (blunted with T4 DNA polymerase)/EcoRI-cut pHB6368, forming Ω-*gfp*-Em^r	
pHB6382a	$Ap^r Cm^r$, pHB247 cut with HpaI, ligated with SmaI-cut pUC19, in the orientation that the zeta plasmid can be excised with KpnI and BamHI	
pHB6382b	Ap^r, *ori* (p15A)-*oriT*(RK2) generated by overlap PCR, using primer pairs pACYC184ori-1/pACYC184ori-oriT-2 and pRL1087bori-oriT-3/pRL1087bori-4, templates pACYC184 and pRL1087b, cloned into pMD18-T	
pHB6444	Ap^r, A DNA fragment (chromosomal bp 2820269-2822306) overlapping *hetR*, with TCCGGA in the upstream region substituted with CAATTG, generated by overlap PCR using primers PhetRcaattg p-1/PhetRcaattg p-2 and PhetRcaattg p-3/PhetRcaattg p-4, cloned into pMD18-T	
pHB6448	$Sm^r Sp^r$, the DNA fragment with TCCGGA substituted, excised with SacI + PstI, cloned into pRL277	
pHB6451	Sm^r/Sp^r, the KpnI-BamHI fragment containing the zeta plasmid excised from pHB6382a, the BamHI-PstI fragment containing *ori*-*oriT* excised from pHB6382b, the PstI-KpnI fragment containing Ω-*gfp*-Em^r excised from pHB6379, ligated into one plasmid	

pHB6458	Sm^r/Sp^r, the minimal DIF$^+$ motif promoter of *patS* generated by annealing oligos patS-1 and patS-2, cloned into the BglII site upstream of *gfp* in pHB6451	
pHB6486	Sm^r/Sp^r, the mutated minimal DIF$^+$ motif promoter (TCCGGA substituted with GATATC) of *patS*, generated by annealing oligos patS-3 and patS-4, cloned into the BglII site upstream of *gfp* in pHB6451	
pHB6821	Sm^r/Sp^r, the minimal DIF$^+$ motif promoter of *hetR* generated by annealing oligos hetRtccgga-1 and hetRtccgga-2, cloned into the BglII site upstream of *gfp* in pHB6451	
pMD18-T	Ap^r, T-vector	TaKaRa
pRL277	Sm^{r}Sp^r, *sacB*-bearing cloning vector	Black et al, 1993
**Primers**	**Sequences (5′→3′)**	**Reference or source**
2-patU3-R	cggaattcctatgttgcgggattaa	This study
alr0101-F	catatgcaagaacgttttcaagcc	
Em-1	ccaaattaaagagggttataatgaacgag	
Em-2	gacctgcatcccttaacttact	
gfp-1	tcagtggagagggtgaaggtga	
gfp-2	cttcgggcatggcactcttga	
HA-tsf-F	gcatatgtacccatacgacgtaccagattacgctatggctgaaattaccgcatcc	
HA-tsf-R	gggatccggtaccaccctggaagtacaggttttcagactgcttggacatcgca	
hetR-race-1	gattacgccaagcttgcatgatctgatccattgcactggggcc	
hetR-race-2	gattacgccaagcttggtcaagatgctcattcctccgt	
hetR-RT-F	gcaccacctagagccaaaac	
hetR-RT-R	ccagacatagggaaactgaatc	
hetRtccgga-1	gatccgttccggataatagggaaagtccttgtaggttacttattagg	
hetRtccgga-2	gatccctaataagtaacctacaaggactttccctattatccggaacg	
HetZ-201-1-F	gtgaaagccgagactatgtctggtgctgtagaaagttca	
HetZ-201-1-R	ctttctacagcaccagacatagtctcggctttcactag	
HetZ-down344-R	actcgaggggctaaccatccccaattagg	

hetZ Mutant-AF	ggtaccggatcccatatgcgccgcgaaaatcaactcgg	
hetZ Mutant-BF	aagcattaaacgctttcactccacgtcagcgagattactt	
hetZ Mutant-BR	gctgacgtggagtgaaagcgtttaatgcttctaaa	
hetZ Mutant-CR	aaactcgagtaaacctaaaacgttctcaatttc	
HetZ-up748-F	gagcgttttctgacatttacaa	
hetZup-RT-F	tacaagttggtatgcgtttag	
hetZup-RT-R	tctcggctttcactagaccct	
Mut-hetZ-F	ggtaccggatcccatatgaagtcagccgcaacagc	
Mut-hetZ-R	aaactcgagttcatgagtggatgcacttgatc	
pACYC184ori-1	ggacatcagcgctagcggagtgt	
pACYC184ori-oriT-2	acgagcaaggcaagaccgatccatacacggtgcctgactgcg	
patU3(MBP)-1	aggatccatgcaagaacgttttcaagcc	
patU3(MBP)-2	agcggccgctgttgcgggattaatga	
pRL1087boriT-ori-3	cgcagtcaggcaccgtgtatggatcggtcttgccttgctcgt	
pRL1087boriT-4	ataagaatgcggccgcttttccgctgcataaccctgcttc	
patS-1	gatccactatccggatttttaccaaataactgtagagaaaagtag	
patS-2	gatcctacttttctctacagttatttggtaaaaaatccggatagtg	
patS-3	gatccactagatatctttttaccaaataactgtagagaaaagtag	
patS-4	gatcctacttttctctacagttatttggtaaaaaagatatctagtg	
patS-RT1	gttctgttgaaaagtaattcac	
patS-RT2	ctaccactaccgcgctcatcac	
pDU1-1	acttccgccgtgctgctgac	
pDU1-2	gttatgggtgacgcggttgtg	
PhetR p-1	ggaaataaaaggtattgctaagttgg	
PhetR p-2	ttactggcgaactttatggttctaataag	
PhetR p-3	ttgctatttttaagacttttaaactgcatttggatttaggagatagcaacac	
PhetR p-4	gtgttgctatctcctaaatccaaatgcagtttaaaagtcttaaaaatagcaa	
PhetR p-5	ttactggcgaactttatggttctaataagtaacctacaaggactttccctattacaattgac ttatctgctctttaatagcg	

PhetRcaattg p-1	cctcatcctgacccgcgtatgtgc	
PhetRcaattg p-2	cctacaaggactttccctattacaattgacttatctgctctttaatagcg	
PhetRcaattg p-3	cgctattaaagagcagataagtcaattgtaatagggaaagtccttgtagg	
PhetRcaattg p-4	ccgctcttggtcgtctgctggggc	
rnpB-RT-1	gcgttggcggttgcagaccagt	
rnpB-RT-2	agtccccagtccccaatcttgg	

[a] Ap, ampicillin; Cm, chloramphenicol; Em, erythromycin; Km, kanamycin; Nm, neomycin; Sm, streptomycin; Sp, spectinomycin; unless stated otherwise, the template for PCR reactions was *Anabaena* sp. genomic DNA.

[b] FACHB, Freshwater Algal Culture Collection of the Institute of Hydrobiology, Chinese Academy of Sciences.

References for Supplementary Table S1

1. Black TA, Cai Y, Wolk CP. Spatial expression and autoregulation of *hetR*, a gene involved in the control of heterocyst development in *Anabaena*. Mol Microbiol. 1993; 9: 77-84.
2. Du Y, Cai Y, Hou S, Xu X. Identification of the HetR-recognition sequence upstream of *hetZ* in *Anabaena* sp. strain PCC 7120. J Bacteriol. 2012; 194: 2297-2306.
3. Ernst A, Black T, Cai Y, Panoff JM, Tiwari DN, Wolk CP. Synthesis of nitrogenase in mutants of the cyanobacterium *Anabaena* sp. strain PCC 7120 affected in heterocyst development or metabolism. J Bacteriol. 1992; 174: 6025–6032.
4. Horton RM, Hunt HD, Ho SN, Pullen JK, Pease LR. Engineering hybrid genes without the use of restriction enzymes: gene splicing by overlap extension. Gene. 1989; 77: 61-68.
5. Rose RE. The nucleotide sequence of pACYC184. Nucleic Acids Res. 1988; 16: 355.
6. Wang Y, Xu X. Regulation by *hetC* of genes required for heterocyst differentiation and cell division in *Anabaena* sp. strain PCC 7120. J Bacteriol. 2005; 187: 8489-8493.
7. Zhang H, Wang S, Wang Y, Xu X. Functional overlap of *hetP* and *hetZ* in regulation of heterocyst differentiation in *Anabaena* sp. strain PCC 7120. J Bacteriol. 2018; 200: e00707-17.
