# Supplementary figures and images for: Expression from DIF1-motif promoters of hetR and patS is dependent on HetZ and modulated by PatU3 during heterocyst differentiation
Source: PLoS One. 2020 Jul 23;15(7):e0232383. doi: 10.1371/journal.pone.0232383 (PMC7377430; doi:10.1371/journal.pone.0232383)

**Fig. 6B**

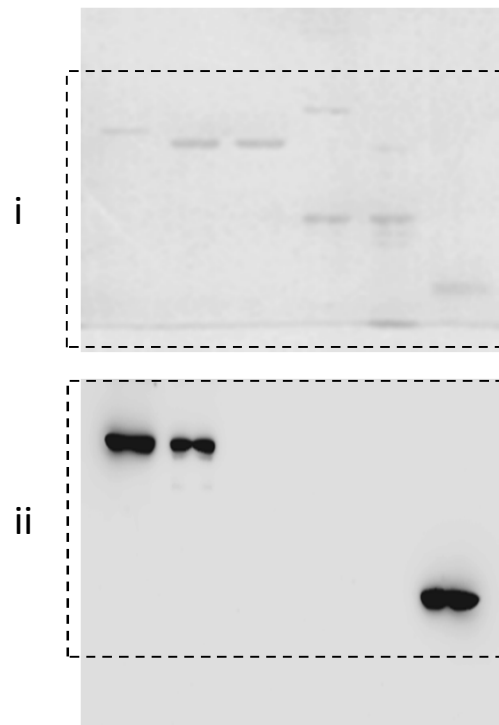

**S5 Fig**

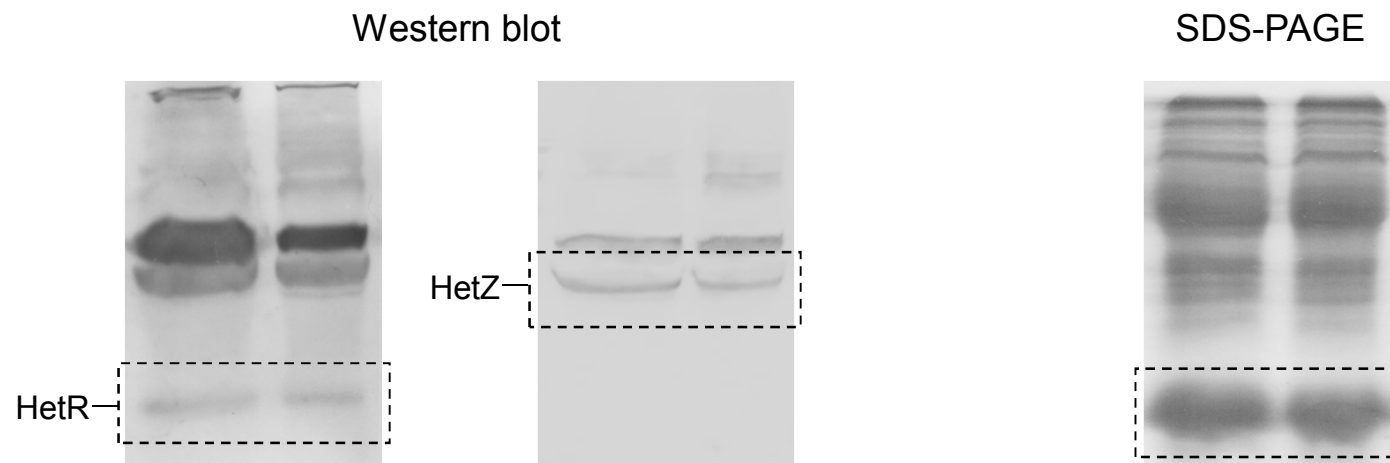

Supplement: S1 Raw images — (PDF) [file pone.0232383.s007.pdf]
